# Mitochondrial Function in Modulating Human Granulosa Cell Steroidogenesis and Female Fertility

**DOI:** 10.3390/ijms21103592

**Published:** 2020-05-19

**Authors:** Dilip Bhargava Sreerangaraja Urs, Wen-Han Wu, Katerina Komrskova, Pavla Postlerova, Yung-Feng Lin, Chii-Ruey Tzeng, Shu-Huei Kao

**Affiliations:** 1 Ph.D. Program in Medical Biotechnology, College of Medical Science and Technology, Taipei Medical University, Taipei 11031, Taiwan; d609107005@tmu.edu.tw (D.B.S.U.); yflin@tmu.edu.tw (Y.-F.L.); 2 School of Medical Laboratory Science and Biotechnology, College of Medical Science and Technology, Taipei Medical University, Taipei 11031, Taiwan; g160093010@tmu.edu.tw; 3 Department of Obstetrics and Gynecology, Taipei Veterans General Hospital, Taipei 11217, Taiwan; 4 Laboratory of Reproductive Biology, Institute of Biotechnology of the Czech Academy of Sciences, BIOCEV, Prumyslova 595, 252 50 Vestec, Czech Republic; Katerina.komrskova@ibt.cas.cz (K.K.); Pavla.Postlerova@ibt.cas.cz (P.P.); 5 Department of Zoology, Faculty of Science, Charles University, Vinicna 7, 128 44 Prague 2, Czech Republic; 6 Department of Veterinary Sciences, Faculty of Agrobiology, Food and Natural Resources, University of Life Sciences Prague, Kamycka 129, 165 00 Prague 6, Czech Republic; 7 Department of Obstetrics and Gynecology, School of Medicine, College of Medicine, Taipei Medical University, Taipei 11031, Taiwan; tzengcr@tmu.edu.tw; 8 Center for Reproductive Medicine, Department of Obstetrics and Gynecology, Taipei Medical University Hospital, Taipei 11031, Taiwan

**Keywords:** granulosa cells, steroidogenesis, estradiol, progesterone, fertilization rate, mitochondrial mass, StAR, 3β-HSD.

## Abstract

Ovarian follicle steroidogenesis associated with embryo quality results in a successful pregnancy. Each follicle consists of an oocyte surrounded by granulosa cells, which secrete several steroid and peptide hormones. Follicles harvested from women who conceived after assisted reproductive therapy (ART) had significantly higher estradiol levels in follicular fluids than the follicles from women who failed to conceive after ART. The higher follicular estradiol levels correlate well with successful fertilization following ART. Mitochondria are the central sites for steroid hormone biosynthesis. The first and rate-limiting step in the biosynthesis of steroid hormones occurs in the mitochondria of granulosa cells. In the present study, we hypothesized that the mitochondria in granulosa cells are critical for maintaining oocyte quality and fertility capacity. This study aims to clarify the relationship between mitochondrial function and granulosa cell steroidogenesis, and the relationship between hormone levels and fertility capacity. Sera, follicular fluids and granulosa cells were obtained from individuals undergoing IVF-ET treatment. The oocyte numbers, oocyte quality, fertilization rate, and pregnancy rate were also recorded. The patients who provided the granulosa cells were further classified into four groups: endometriosis, ovarian endometrioma, endometriosis without ovarian endometrioma, and polycystic ovary syndrome (PCOS); patients with other female factor infertility and male factor infertility were used as controls. We measured the levels of estradiol (E2) by radioimmunoassay. Concurrently, we analyzed the mitochondrial mass and membrane potential, and apoptosis by flow cytometry using nonyl acridine orange, TMRE, Annexin V-FITC and PI. Mitochondrial morphology was visualized by transfection with pLV-mitoDsRed. In addition, we assessed the protein levels of steroidogenic enzymes, steroidogenic acute regulatory protein (StAR) and 3β-hydroxysteroid dehydrogenase (3β-HSD) by Western blot. The results showed significantly decreased serum E2 and follicular E2 levels, and decreased IVF outcomes, in the patients with endometriosis. Reduced mitochondrial mass and decreased mitochondrial membrane potential were correlated with lower E2. Furthermore, a significant decrease in StAR and 3β-HSD was found in patients with ovarian endometrioma. The enzyme levels of StAR and 3β-HSD were highly correlated with E2 levels. Finally, elevated cumulus cell apoptosis was found in the patient group with ovarian endometrioma and PCOS. In conclusion, mitochondrial dysfunction of human granulosa cells may contribute to the decline of steroidogenesis, decreased fertilization rate, oocyte maturation rate, and oocyte quality, and it can ultimately jeopardize fertility.

## 1. Introduction

Recently, an increasing number of infertile women and those who postpone childbearing are requesting the aid of assisted reproductive technology to reproduce. Infertility is the inability to achieve pregnancy for more than 12 months of unprotected sexual intercourse, and it affects many women throughout the world [1]. It is expected that live birth rates improve after assisted reproductive techniques, such as artificial insemination (IUI), in vitro fertilization (IVF), intracytoplasmic sperm injection (ICSI) and embryo transfer (ET). Infertile patients receive comprehensive infertility evaluation to determine the infertility causes, including history and physical examination, diagnosis tests including semen analysis, documentation of normal ovulatory function and tubal occlusion, ovarian reserve testing [day 3 follicle-stimulating hormone (FSH), estradiol (E_2_), clomiphene citrate challenge test, anti-Müllerian hormone (AMH), or antral follicle count], and the assessment of fallopian tube patency and uterine cavity [2,3,4].

If the conventional tests fail to reveal any apparent cause of infertility, then the condition is labeled as unexplained infertility [5]. Male factor infertility is defined as one or more abnormal semen parameters detected on semen analysis, or the presence of inadequate sexual or ejaculatory function [6]. Ovulation is most easily addressed by a mid-luteal phase serum progesterone level (>3 ng/mL Endometriosis is characterized by the outgrowth of endometrial glands and stroma in sites other than the uterine cavity, most commonly in the pelvic cavity, ovaries, uterosacral ligaments and the pouch of Douglas [7,8,9]. Endometriosis is an estrogen-dependent, non-malignant and inflammatory disease that affects women during their premenarcheal, reproductive and postmenopausal hormonal stages [10]. Endometriosis is surgically staged according to the revised American Society for Reproductive Medicine scoring system, from the minimal Stage I to the severe Stage IV. Ovarian endometrioma is characterized as a severe Stage IV disease, and is formed when ectopic endometrial tissue within the ovary bleeds and results in a hematoma surrounded by ovarian parenchyma [11]. Polycystic ovary syndrome (PCOS) is a prevalent hormonal disorder of premenopausal women worldwide, and is characterized by hirsutism, hyperandrogenemia, polycystic ovaries, oligo-ovulation, irregular or absent menstrual cycles, and infertility [12,13]. Based on several data points, the distribution of primary diagnoses are as follows: unexplained (26%); abnormal semen parameters (24%); tubal disease (23%); ovulatory disorders (18%); endometriosis (5% to 10%); polycystic ovary syndrome (PCOS, 6% to 8%); and other female factors (3%) [4]. 

The quality of gametes, embryos and maternal environment for embryo implantation are the crucial parameters in the ability to achieve a pregnancy or live birth. Accumulated evidence shows orchestrated cross-talk between follicular somatic cells (granulosa cells and theca cells) and oocytes [14]. In addition, cumulus and granulosa cells may be used to gain insight into the viability and reproductive potential of oocytes [15]. Specifically, cumulus oophorus cells (called cumulus cells) accompanying ovulated oocytes are also capable of secreting many cytokines, growth factors, and steroids, that may be important in oocyte nourishment [16]. Both granulosa and theca cells are steroid-producing cells. Theca cells synthesize progesterone (P4) and androgens, which serve as precursors for estrogen synthesis in granulosa cells [17]. Estradiol (E2) improves follicle survival, growth, antrum formation, and oocyte health, whereas P4 provides few beneficial effects on follicle survival [18].

Steroidogenic acute regulatory protein (StAR) and 3-beta-hydroxysteroid dehydrogenase (HSD3β1) are key factors involved in steroidogenesis, and are significantly associated with oocyte quality and pregnancy outcome [19,20,21]. It is well known that mitochondria are critical sites for steroid hormone biosynthesis. The initiated and rate-limiting step in the biosynthesis of steroid hormones is the transfer of cholesterol into mitochondria, which is facilitated by StAR [22]. Therefore, functional mitochondria with an intact mitochondrial membrane potential were demonstrated to facilitate granulosa cell steroidogenesis [22,23]. Studies have reported that the uncoupler carbonyl cyanide m-chlorophenyl hydrazone (CCCP) disrupts mitochondrial membrane potential and prevents StAR from being transported into the mitochondrial matrix [24,25]. In addition, the addition of Fo/F1 adenosine triphosphate (ATP) inhibitor (oligomycin) to the cells results in a decrease in ATP synthesis, StAR protein expression, and P4 production [24]. Furthermore, studies demonstrated that the occurrence of mitochondrial genome mutations in granulosa cells, such as the mitochondrial DNA (mtDNA) 4977 bp deletion, was associated with granulosa cell apoptosis [26].

Mitochondria have been demonstrated as key factors controlling female reproductive processes. Degenerated granulosa cells and cumulus cells have been shown in relation to mitochondrial swelling, leading to cell apoptosis and follicle atresia [27]. The study revealed that reduced granulosa cell counts, decreased cell viability and enhanced mitochondrial anomalies in human granulosa (cumulus) cells were found in the patients with endometriosis and PCOS [28]. Recent investigations have shown mitochondrial dysfunction and mutation in the granulosa (cumulus) cells of the patients with endometriosis [29] or PCOS [12]. The granulosa (cumulus) cells are the critical and important sensors for follicle and oocyte health. The work reported here examines the hypothesis that mitochondria in granulosa are critical for maintaining oocyte quality and fertility capacity. To understand whether impaired steroidogenesis leads to decreased ART outcome, we analyzed the relationship between the oocyte maturation rate, oocyte quality, fertilization rate and pregnancy rate with estradiol or progesterone levels, in patients with endometriosis, PCOS, male factor or other female infertility factors. We further analyzed the relationship between mitochondrial function and steroidogenic enzymes (StAR and 3β-HSD). Here, we highlight the fact that mitochondrial dysfunction of human granulosa cells may contribute to the global decline of steroidogenesis, oocyte maturation rate, and fertilization rate, and it can ultimately jeopardize fertility.

## 2. Results

### 2.1. A Decreased Fertilization Rate and Pregnancy Outcome were Found in the Patient Group with Ovarian Endometriotic Cyst

The fertilization rate was calculated as the number of oocytes fertilized out of the number of mature oocytes inseminated. The pregnancy rate was the success rate for becoming pregnant. Infertile individuals were characterized and grouped into six groups: other female factors (female factor without endometriosis and PCOS), endometriosis (combined the groups with ovarian endometrioma and the endometriosis without ovarian endometrioma), ovarian endometrioma, endometriosis without ovarian endometrioma, male factor, and polycystic ovary syndrome. The average ages and patient numbers were 36 ± 3.5 (*n* = 75 in other female factor); 36 ± 3.9 (*n* = 81 in endometriosis); 36 ± 3.7 (*n* = 41 in ovarian endometrioma); 36 ± 3.9 (*n* = 40 in endometriosis without ovarian endometrioma); 32 ± 4.1 (*n* = 30 in male factor); 36 ± 3.8 (*n* = 59 in PCOS).

**Figure 1:**
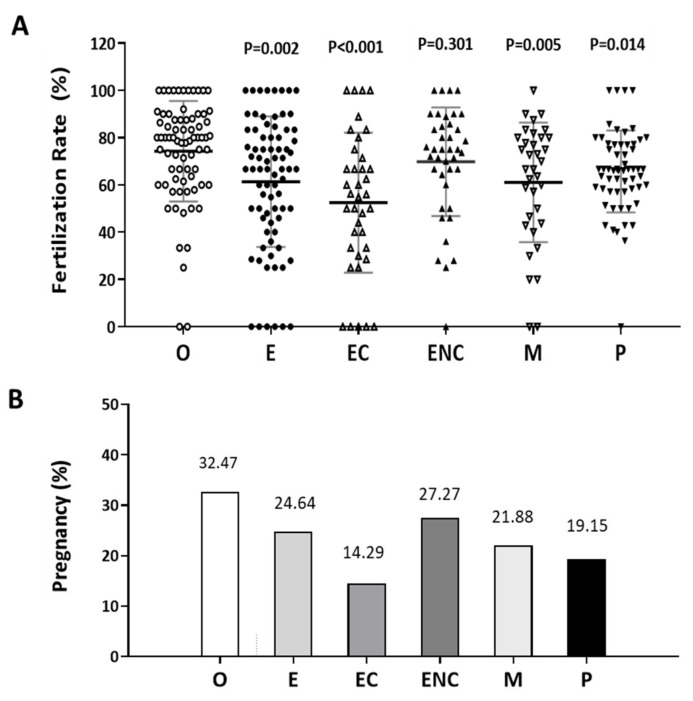
Comparison of the pregnancy and fertilization in the different groups as an indication of infertility. The (**A**) fertilization rate and (**B**) pregnancy rate in each group were counted. The infertile individuals were characterized into six groups: other female factors (O, female factor without endometriosis and PCOS), endometriosis (E), ovarian endometrioma (EC), endometriosis without ovarian endometrioma (ENC), male factor (M), and polycystic ovary syndrome (P). The fertilization rate and pregnancy outcome values were significantly lower in the EC group than they were in the other groups. The *p*-value for each group versus the group with other female factors was determined by an unpaired *t*-test.

In these two analyses, the other female factor group was used as the control group. Except for the male factor group treated by intracytoplasmic sperm injection (ICSI), all the other groups were conventionally inseminated by IVF. Compared to the other female factor group, we found the fertilization rate to be significantly lower in the endometriosis, ovarian endometrioma, and PCOS groups (Figure 1A). In addition, we found that the pregnancy rate of each group was lower than that of the other female group (32.47%, *n* = 75). The pregnancy rates were 24.67% (*n* = 76), 14.29% (*n* = 16), 27.27% (*n* = 38), 21.88% (*n* = 28), and 19.15% (*n* = 49) in the endometriosis, ovarian endometrioma, endometriosis without ovarian endometrioma, male factor, and PCOS groups, respectively (Figure 1B).

### 2.2. Decreased IVF Outcomes were Found in the Various Infertile Groups

**Figure 2:**
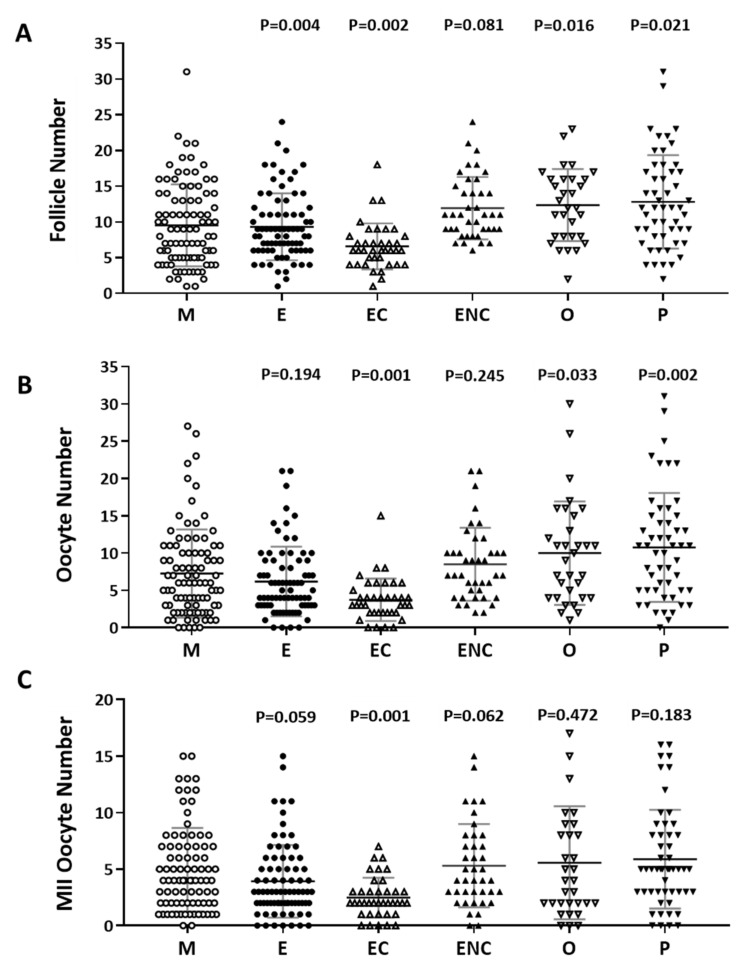
Comparison of oocyte quality and quantity in the different groups as an indication of infertility. The (**A**) follicle number, (**B**) oocyte number, and (**C**) number of mature oocytes (metaphase II) from all infertility patient groups. The infertile individuals were grouped into six groups: male factor (as control group, M), endometriosis (E), ovarian endometrioma (EC), endometriosis without ovarian endometrioma (ENC), other female factors (O), and polycystic ovary syndrome (P). The *p*-value for each group versus the male factor group was determined by an unpaired *t*-test.

The number of follicles, retrieved oocytes and mature metaphase II oocytes were counted and compared. The male factor group was used as a control group. We found significantly decreased follicle numbers, retrieved oocyte numbers, and mature oocytes in patients with endometriosis or ovarian endometrioma (Figure 2). We found that the poorest IVF outcomes were in the ovarian endometriotic cyst group.

### 2.3. A Positive Correlation was Found between Serum Estradiol and Cycle Follicle Outcomes

**Figure 3:**
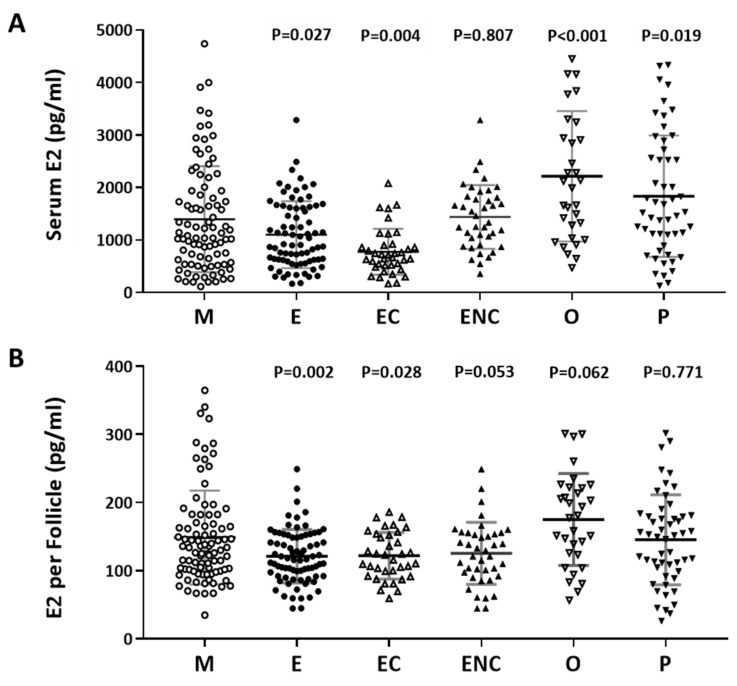
The relationship between serum estradiol (E2) and cycle follicle outcomes in the various infertile groups. (**A**) Serum from each infertile group was collected, and serum E2 (pg/mL) was analyzed by radioimmunoassay (RIA). (**B**) Serum E2 per follicle in each group was compared. The E2 content in the serum before removing the eggs was divided by the number of follicles. The *p*-value for each group versus the male factor group was determined by an unpaired *t*-test.

Furthermore, we investigated the relationship between serum estradiol (E2) and cycle follicle outcomes in the various infertile groups. Individuals who presented with ovarian hyperstimulation syndrome were excluded from the data analysis. The serum E2 level before oocyte retrieval and the E2 level in each follicle was analyzed. Decreased levels of estradiol were observed in the endometriosis, ovarian endometrioma, endometriosis without ovarian endometriotic cysts, and other female factor groups (Figure 3A). The serum concentration was divided by the number of retrieved follicles, and a lower quotient of E2 versus the follicle number was found in the patients with endometriosis, ovarian endometrioma, endometriosis without ovarian endometriotic cysts, and other female factors (Figure 3B). We found that the lowest response to IVF treatment was found in the ovarian endometriotic cyst group.

### 2.4. Serum Progesterone Was Positively Correlated with Follicle Cycle Outcomes

**Figure 4:**
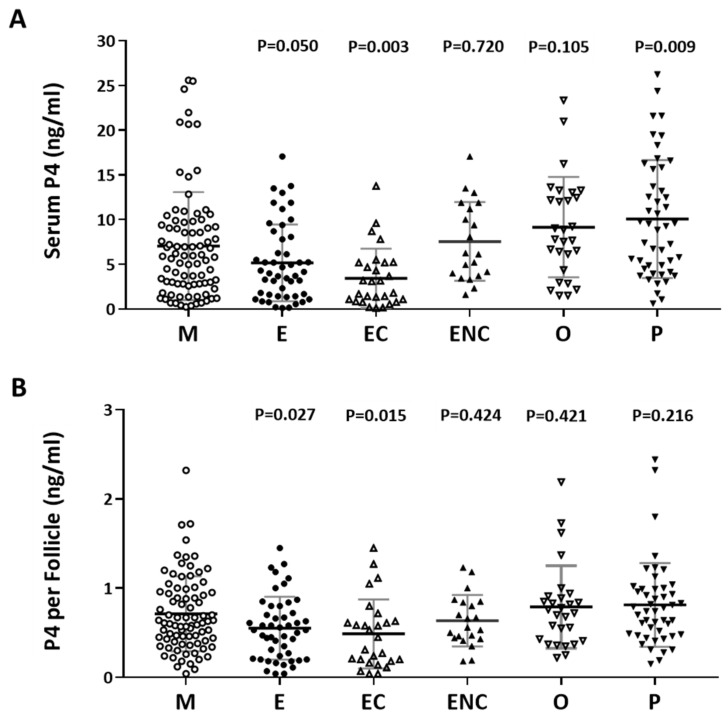
The relationship between serum P4 and cycle follicle outcomes in the various infertile groups. (**A**) Serum from each infertile group was collected, and serum P4 was analyzed by radioimmunoassay (RIA). (**B**) The serum P4 per follicle was compared in the various infertile groups of each patient group. The *p*-value for each group versus the male factor group was determined by an unpaired *t*-test.

The content of progesterone (P4) after oocyte retrieval was measured. Individuals who presented with ovarian hyperstimulation syndrome were excluded from the data analysis. The serum P4 level before oocyte retrieval and the P4 in each follicle were analyzed. Decreased P4 levels were observed in the endometriosis, ovarian endometrioma, and other female factor groups (Figure 4A). The serum concentration was divided by the number of retrieved follicles, and a lower quotient of P4 versus the follicle number was found in the patients with endometriosis and ovarian endometrioma (Figure 4B). We found that a significant decrease in progesterone was found in the ovarian endometriotic cyst group.

### 2.5. Decreased E2 Content in Follicular Fluids were Found in the Patient Group with Ovarian Endometrioma

**Figure 5:**
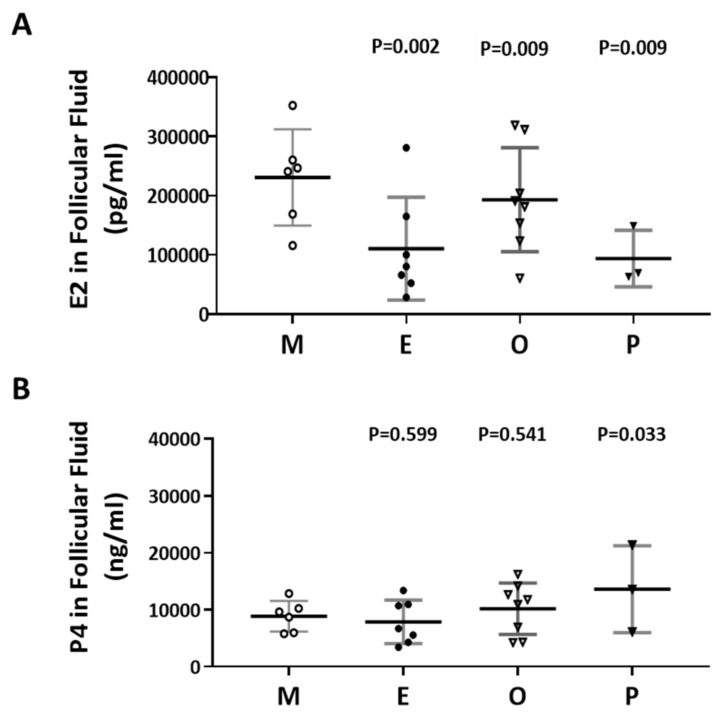
Comparison of the levels of E2 and P4 in the follicular fluid of the various infertile groups. (**A**) E2 content in the follicular fluid. (**B**) P4 content in the follicular fluid. The follicular fluid of the patient was collected, and the contents of E2 (pg/mL) and P4 (ng/mL) in the follicular fluid were analyzed by radioimmunoassay (RIA). The *p*-value for each group versus the male factor group was determined by an unpaired *t*-test.

The levels of E2 and P4 in the follicular fluid from the various infertile groups were examined (Figure 5A,B). The E2 content of the endometriosis group was 52.2% lower than that of the male factor group (110415 ± 32798 vs 230815 ± 33189, *p* = 0.002). Decreased E2 levels in follicular fluid were found in the endometriosis and PCOS groups (Figure 5A). However, increased P4 levels in follicular fluid were revealed in the PCOS group, and no significant difference was observed in the other groups (Figure 5B). The P4 content in the PCOS group was 54.7% higher than that in the male factor group (13625 ± 4394 vs 8866 ±1098, *p* = 0.033). 

### 2.6. Impaired Mitochondrial Function in Cumulus Cells was Found in the Various Infertile Groups

**Figure 6:**
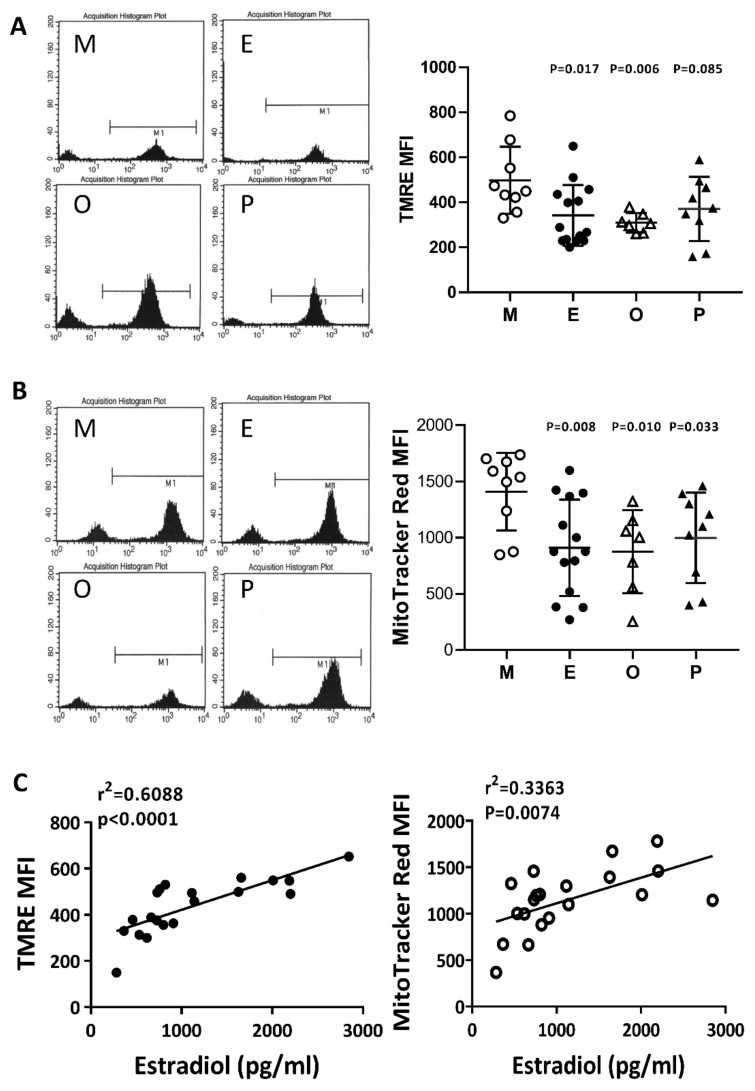
Comparison of mitochondrial membrane potential in the various infertile groups. The mitochondrial membrane potential was measured by (**A**) N, N, N’, N’-tetramethyl-ethylenediamine (TMRE) and (**B**) MitoTracker Red staining and measured by flow cytometry. (**C**) The relationships between the mitochondrial membrane potential (TMRE or MitoTracker Red) and the serum E2 content were assessed by linear regression.

The membrane potential in human cumulus cells was characterized by two specific fluorescent dyes (N, N, N’, N’-tetramethylethylenediamine; TMRE) and MitoTracker Red by flow cytometry analysis. In the present study, we concurrently identified a positive selection marker, the FSH receptor, which is a specific marker for cumulus cells. The evaluation of mitochondrial membrane potential was performed with TMRE or MitoTracker Red, where the high potential is indicated by strong fluorescent intensity. Compared with the male factor group, the membrane potential was significantly decreased in the patients with endometriosis or other female factors (Figure 6A,B). Following MitoTracker Red staining, a significant decrease in mitochondrial membrane potential was found in the PCOS group. On the other hand, if the disease groups showed no change, then the mitochondrial membrane potential change and the patient’s E2 content are statistically analyzed. The results showed that the mitochondrial membrane potential was positively correlated with E2 content (r^2^ = 0.6088, *p* < 0.0001 by TMRE staining, and r^2^ = 0.3363, *p* < 0.0074 by MitoTracker Red staining) (Figure 6C).

### 2.7. Reduced Mitochondrial Mass in Cumulus Cells was Found in the Various Infertile Groups

**Figure 7:**
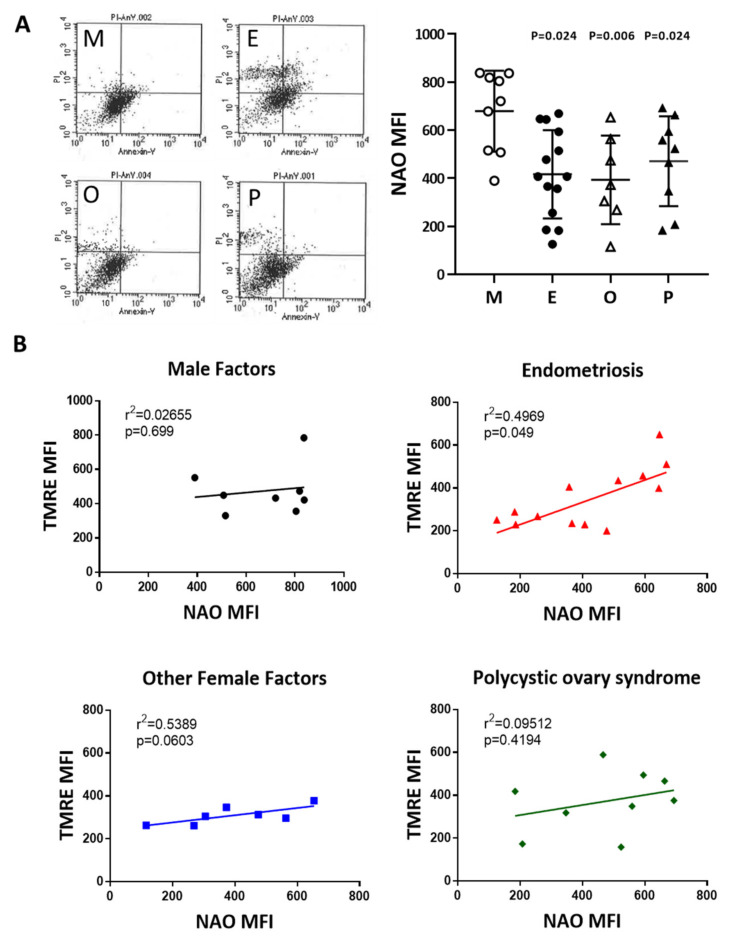
Comparison of mitochondrial mass in the various infertile groups. (**A**) The mitochondrial mass was measured by nonyl acridine orange (NAO) staining and flow cytometry. Reduced mitochondrial mass in cumulus cells was found in patients with endometriosis, other female factors, and PCOS. (**B**) The relationship between the mitochondrial potential and mitochondrial mass in the various groups was analyzed.

To compare mitochondrial mass in cumulus cells, nonyl acridine orange (NAO) was used in flow cytometry analysis. Compared with the male factor group, the fluorescence intensity of each group was low; taking NAO as an example, the intensity in the endometriosis group was 61.4% of that of the male factor group (416.7 ± 49.1 vs 679.2 ± 56.2, *p* = 0.024), and the other female infertility factors were 57.9% of that of the male factor group (393.4 ± 69.8 vs 679.2 ± 56.2, *p* = 0.006). Compared with the male factor group, polycystic ovary syndrome was 67.9% of the male factor group (470.9 ± 62.3 vs 679.2 ± 56.2, *p* = 0.024), and each group was significantly different (Figure 7A). The relationship between the mitochondrial potential and mitochondrial mass in the various groups was analyzed in Figure 7B. The mitochondrial membrane potential was positively correlated to the mitochondrial mass in the four groups.

### 2.8. Induced Mitochondrial Fragmentation and Constrained Cumulus Cell Expansion were Found in the Endometriosis and PCOS Group

**Figure 8:**
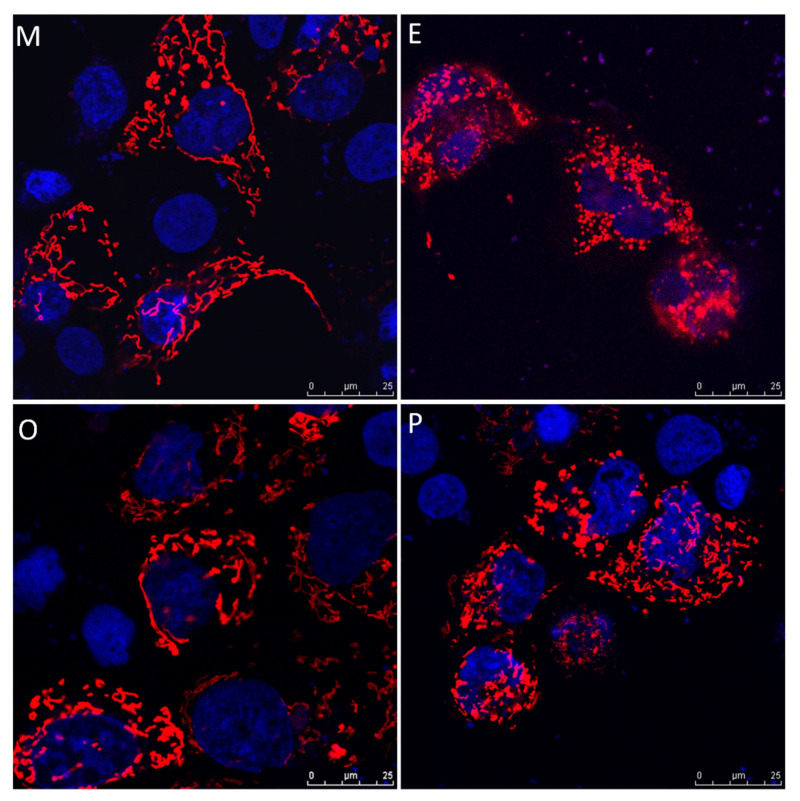
Representative immunofluorescent images for mitochondrial morphology in male factor (**M**), endometriosis (**E**), other female factors (**O**), and polycystic ovary syndrome (**P**). Images of cumulus cells from in vitro fertilization (IVF) patients were analyzed by confocal microscopy to visualize the mitochondrial network and cumulus cell expansion. Representative images show the fragmentation of the mitochondria in the endometriosis group and PCOS group. Scale bar = 25 µm.

Human cumulus cells were collected from the various groups, and were transfected with pLV-mitoDsRed and visualized by confocal microscope. The cumulus cells exhibited higher mitochondrial mass, mitochondrial tabularization and cell expansion in the male factor group (M) and other female factors group (O). Reduced mitochondrial mass, mitochondrial fragmentation and constrained cell expansion were observed in the endometriosis group (E) and PCOS group (P) (Figure 8).

### 2.9. Lower Levels of Steroidogenic Enzymes were Observed in the Various Infertile Groups

**Figure 9:**
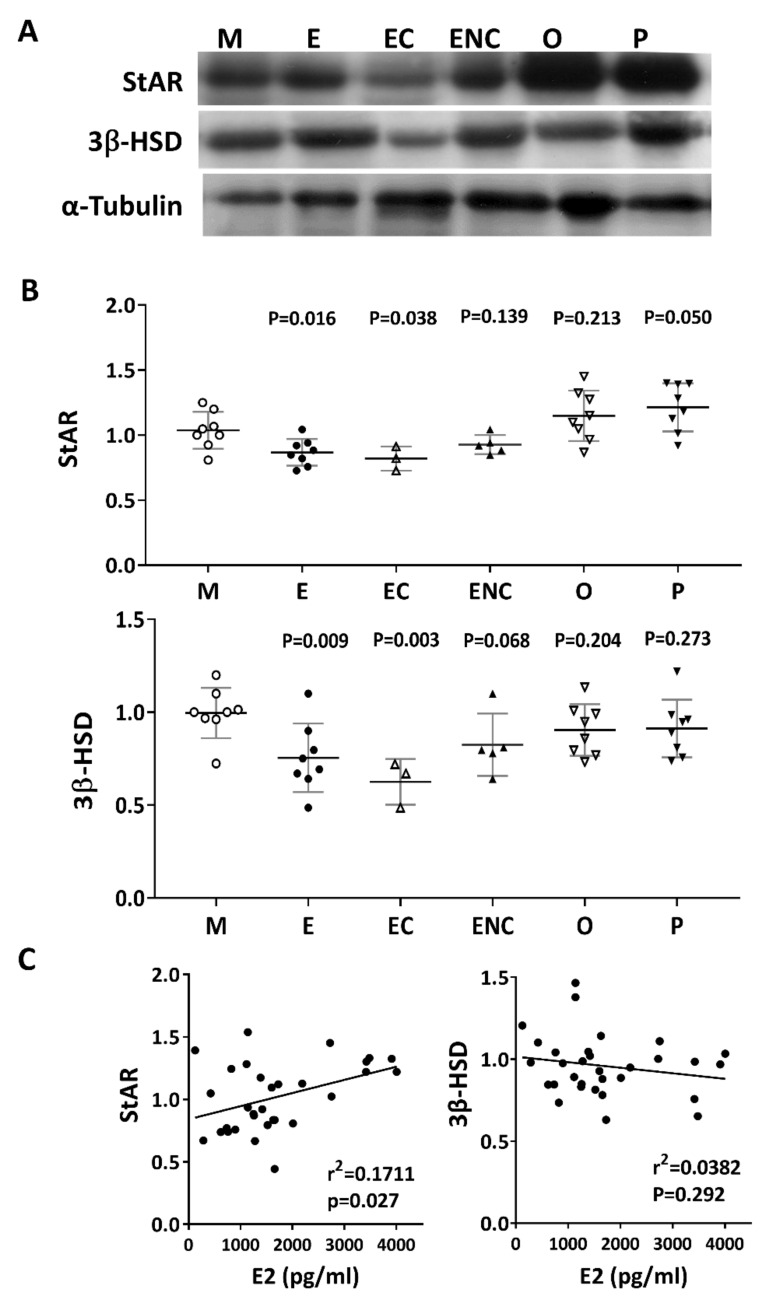
Alteration of key steroidogenic enzymes in various infertile groups. Steroidogenic acute regulatory protein (StAR) and 3 beta-hydroxysteroid dehydrogenase (3β-HSD) are key steroidogenic enzymes. (**A**) Representative immunoblots reveal StAR (30 kDa) and 3β-HSD (43 kDa) levels in granulosa cells, as determined by Western blot; α-tubulin (50 kDa) was used as an internal control. (**B**) Data quantification of StAR and 3β-HSD. (**C**). Linear regression analysis was performed on serum E2 content with StAR or serum E2 content with 3β-HSD in granulosa cells.

Steroidogenic acute regulatory protein (StAR) and 3 beta-hydroxysteroid dehydrogenase (3β-HSD) are key steroidogenic enzymes. Representative immunoblots of StAR and 3B-HSD from human cumulus cells are shown in Figure 9A. Compared with the male factor group, a significant decrease in StAR (76.8% of male factor) and 3β-HSD (61.3% of male factor) was found in the patients with ovarian endometrioma. Conversely, increased levels of StAR and 3β-HSD were detected in the other female factor and PCOS groups (Figure 9B). On the other hand, if the disease group was not different, the levels of StAR and 3β-HSD and the patient’s E2 content were statistically analyzed. The results showed that StAR was positively correlated with E2 content (r^2^ = 0.1711, *p* < 0.027), and 3β-HSD was negatively correlated with E2 content (r^2^ = 0.382, *p* = 0.292) (Figure 9C).

### 2.10. Induced Cumulus Cell Apoptosis was Found in the Patient Group with Ovarian Endometrioma and PCOS

**Figure 10:**
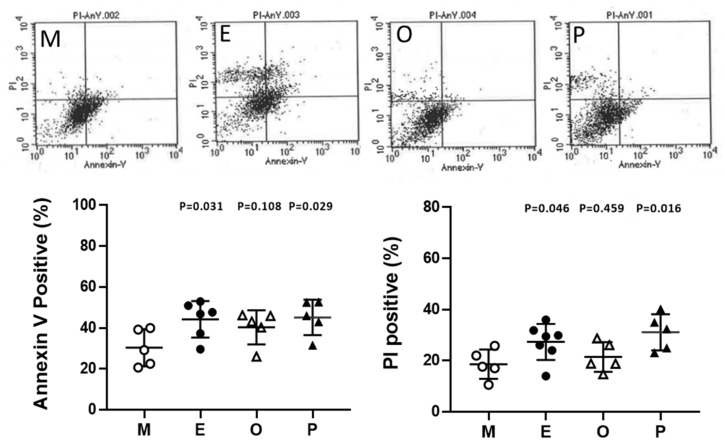
Alteration of granulosa cell apoptosis in various groups. Apoptosis was measured by staining the cell membrane with annexin V- fluorescein-5-isothiocyanate FITC, staining the nucleus with propidium iodide (PI), and measuring the proportion of Annexin V-positive or PI-positive cells by flow cytometry. The top panel shows a scatter plot of each group [male factor (**M**), endometriosis (**E**), other female factors (**O**), and polycystic ovary syndrome (**P**)] in a flow cytometer. The bottom panel shows quantitative flow cytometry data. The *p*-value for each group versus the male factor group was determined by an unpaired *t*-test.

Cumulus cells were stained with annexin V-conjugated fluorescein-5-isothiocyanate (FITC) and propidium iodide (PI) and then were analyzed by flow cytometry. In the present analysis, the male factor infertility condition was considered the male factor group, and each group showed a higher proportion of cell death compared to the male factor group. Apoptosis levels within cumulus cells were significantly increased in patients with endometriosis and PCOS. Taking Annexin V positive as an example, the endometriosis group had 45.8% higher levels than the male factor group (44.2% ± 3.7% vs 30.3% ± 4.1%, *p* = 0.031). A 1.6-fold and 1.7-fold increase in PI-positive cumulus cells was found in endometriosis and PCOS, respectively (Figure 10).

## 3. Discussion

**Figure 11:**
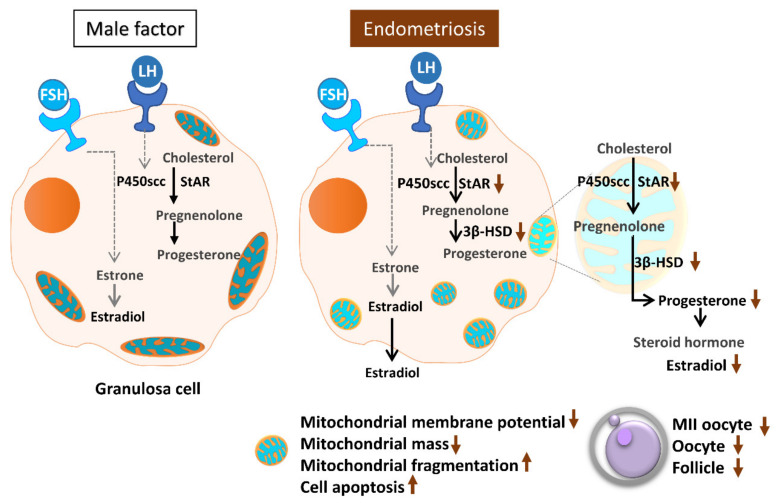
Schematic diagram summarizing the mitochondrial function in modulating human granulosa cell steroidogenesis and female fertility. Mitochondrial dysfunction of human granulosa cells may contribute to the decline of steroidogenesis, decreased fertilization rate, oocyte maturation rate, and oocyte quality, and it can ultimately jeopardize fertility.

Assisted reproductive technology helps many infertile patients who would otherwise be unable to conceive. However, infertility treatment still faces numerous challenges and problems, most notably the low success rate and consequent repeated implantation failure. In essence, many factors can contribute to this problem, most of which are due to the nature of the human reproduction and fertilization processes. Oocyte quality is known to be a unique and pivotal factor involved in successful reproduction [14,30]. Cumulus cells are critical for oocyte development, maturation, ovulation and fertilization [31]. Meanwhile, a highly active mitochondrial metabolism in cumulus cells supports the energy that cumulus–oocyte complexes require to achieve a successful pregnancy [32,33]. In the present study, we evaluated mitochondrial function in human granulosa cells from infertile patients with various clinical backgrounds. Various mitochondrial anomalies have been linked to the endometriosis group. In the present study, a significant decrease of E2 levels in serum and follicular fluid and decreased IVF outcomes were observed in patients with endometriosis. In the collected cumulus cells, reduced mitochondrial mass and decreased mitochondrial membrane potential were correlated with lower serum E2 levels. Furthermore, the enzyme levels of StAR and 3β-HSD were correlated with serum E2 levels. Finally, elevated cumulus cell apoptosis was found in the patient group with ovarian endometrioma and PCOS (Figure 11).

Accumulating reports indicate that endometriosis is associated with lower numbers of harvested oocytes, lower implantation rates, and lower pregnancy rates after IVF [34,35,36]. Some studies have shown that endometriosis affects oocyte yield, but not embryo quality or pregnancy outcome, regardless of the presence of an ovarian endometrioma [37,38]. In this study, the fertilization rate, pregnancy outcome and IVF outcome obtained from the endometriotic group were significantly lower than those of the other groups (*p* < 0.05). In particular, the patients with ovarian endometrioma had the lowest number of follicles, and compared to other groups, the number of eggs obtained and their maturity was lowest in that cohort. 

The local intrafollicular niche of patients might impact oocyte quality. Follicular fluid is a distinct microenvironment within the ovaries that promotes follicle maturation and oogenesis [39,40]. In addition, there is decreased cytochrome P450 aromatase expression and increased reactive oxygen species generation in the follicular dynamic environment [41]. Damaging oxidative stress and an inflammatory environment have also been deduced as causes of diminished oocyte quality [41]. Several studies have reported that E2 and P4 attenuate oxidative insults induced by environmental chemicals [42]. Steroid hormones are synthesized in granulosa cells and theca cells of the ovary. In normal fertile individuals, elevated levels of E2 and P4 were found in follicular fluids in which oocytes with higher fertilization rates and pregnancy rates were found [43,44]. In addition, lower serum estradiol at the time of human chorionic gonadotropin (HCG) administration has been demonstrated to be a predictor of poor IVF outcome and pregnancy rates [45,46]. 

In the present study, the patients received the controlled ovarian stimulation with exogenous gonadotropins by long gonadotropin-releasing hormone (GnRH) agonist protocol, and we monitored the levels of serum estradiol and the responsive follicle numbers during hormone stimulation. The ovulation was triggered when follicles reached >17 mm in diameter by HCG. Each follicle with a diameter of >10 mm was aspirated. However, no oocyte was retrieved from some small follicles. Moreover, the level of steroid secretion (E2) is correlated to the size of follicles [47]. In the present study, the follicular fluids were harvested from the larger follicles (≥ 20 mm). E2 and P4 in the blood were obtained 10 min before the administration of HCG. We found that serum estradiol was positively correlated with follicle outcomes in the IVF cycle. The serum concentration was divided by the number of retrieved follicles, and a lower quotient of estradiol versus the follicle number was found in the patients with endometriosis, ovarian endometrioma, endometriosis without ovarian endometriotic cysts, or other female factors. Only patients with polycystic ovary syndrome had higher E2 levels, and there was no significant difference from the male factor group. In addition, a lower quotient of serum P4 versus follicle number was found in patients with endometriosis and ovarian endometrioma. However, the quotients of E2 and P4 in follicular fluid versus follicle numbers were found to be lower and higher, respectively, in the PCOS group than they were in other groups.

**Figure 12:**
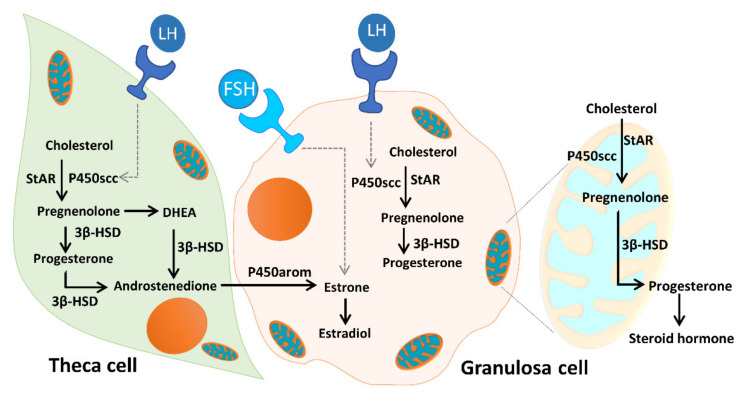
Mitochondrial function in modulating human granulosa cell steroidogenesis and female fertility. Cholesterol transferred from granulosa cells, by low-density lipoprotein (LDL) receptor-mediated endocytosis, into theca cells, where it is used as a substrate for steroidogenesis. The conversion of cholesterol to pregnenolone is initiated by the binding of luteinizing hormone (LH) to the LH receptor (LHR), and subsequent conversion of androgens to E2 is initiated by the binding of follicle-stimulating hormone (FSH) to the follicle-stimulating hormone receptor (FSHR). Mitochondria are the central sites for steroid hormone biosynthesis. The first step in the biosynthesis of steroid hormones is the transfer of cholesterol to the mitochondrial outer membrane, which is facilitated by StAR. Then, cytochrome P450scc (CYP11A1) initiates steroidogenesis by converting cholesterol to pregnenolone at the mitochondrial inner membrane, and the enzyme 3β-HSD binds with P450scc to form a complex inserted into the mitochondrial inner membrane of the mitochondria to synthesize progesterone. The mitochondrial intermembrane proton gradient is essential for the 3β-HSD activity. Mitochondrial dysfunction of the human granulosa cells may contribute to the decline of steroidogenesis.

As Figure 12 illustrates, unesterified cholesterol can be deposited from granulosa cells by low-density lipoprotein (LDL) receptor-mediated endocytosis into theca cells, where it is used as a substrate for steroidogenesis. The conversion of cholesterol to pregnenolone is initiated by the binding of luteinizing hormone (LH) to the LH receptor (LHR), and subsequent conversion of androgens to E2 is initiated by the binding of FSH to the FSHR. Mitochondria are the central sites for steroid hormone biosynthesis. The first and rate-limiting step in the biosynthesis of steroid hormones is the transfer of cholesterol to the mitochondrial outer membrane, which is facilitated by StAR [22]. Then, cytochrome P450scc (CYP11A1) initiates steroidogenesis by converting cholesterol to pregnenolone at the mitochondrial inner membrane. Cytochrome P450 type I enzymes, CYP11A1, CYP11B1, CYP11B2, CYP24A1, CYP27A1, CYP27B1, and CYP27C1, are the seven P450 enzymes exclusively located in mitochondria [22,23,48]. In addition, the enzyme 3β-HSD binds with P450scc, to form a complex inserted into the mitochondrial inner membrane of the mitochondria to synthesize progesterone. The mitochondrial intermembrane proton gradient is essential for 3β-HSD activity [49]. Respiratory competent mitochondria are required to facilitate steroidogenesis in granulosa cells [22,23]. In the present study, we evaluated the mitochondrial function of cumulus cells in infertile patients. We found that the mitochondrial membrane potential and mitochondrial mass were positively correlated with E2 content. The enzyme contents of StAR and 3β-HSD were highly correlated with E2 levels. Moreover, we found a significant decrease in StAR and 3β-HSD in patients with ovarian endometrioma. Conversely, increased levels of StAR and 3β-HSD were detected in the PCOS groups.

A previous study showed that mitochondrial dynamics might be pivotal for steroidogenesis. Interruption of mitochondrial fusion by knocking down Mfn2 expression causes a negative impact on steroidogenesis [50]. In response to the LH surge, cumulus cell expansion of the cumulus–oocyte complex is necessary for oocyte maturation [32]. We demonstrated that an ER antagonist (ICI 182,780) and oligomycin (a mitochondrial respiratory inhibitor) disrupted the mitochondrial dynamic network and constrained the expansion of cumulus cells. This indicated that estradiol might contribute to maintaining the dynamic mitochondrial network. We found that a mitochondrial respiratory inhibitor (oligomycin) restricted expansion. Hence, mitochondria function to support steroidogenesis and granulosa cell differentiation.

Granulosa cells are essential for follicular growth and maintenance, and play a major role in deciding the survival of follicles. A recent study demonstrated that elevated granulosa cell apoptosis within the maturing ovarian follicle reflects ovary aging. Infertile patients with poor prognosis represent a greater incidence of apoptosis [51]. In our previous findings, we showed that a higher occurrence of mitochondrial DNA (mtDNA) mutations in granulosa cells, such as the mtDNA 4977 bp deletion, was associated with granulosa cell apoptosis [26]. In this study, our data showed that there was more cumulus cell apoptosis found in the patient group with ovarian endometrioma and PCOS than there was in other groups of infertile patients. 

## 4. Materials and Methods

### 4.1. Sample Collections

Serum, follicular fluids and granulosa cells were obtained from patients who received IVF therapy at the Department of Obstetrics and Gynecology of Taipei Veterans General Hospital and the Department of Gynecology and Obstetrics, Taipei Medical University Hospital. This study was performed according to the tenets of the Declaration of Helsinki for research involving human subjects. All specimens were collected according to the protocol immediately after follicle aspiration, and approval was received from the Institutional Review Board of Taipei Veterans General Hospital (97-02-43) and Taipei Medical University Hospital (TMU-JIRB N20190717). The infertile individuals were characterized and organized into six groups: other female factors (including pelvic inflammation, tubal obstruction or unknown causes), endometriosis, ovarian endometrioma (chocolate cysts), endometriosis without ovarian endometrioma, male factor, and polycystic ovary syndrome (PCOS). 

All women underwent IVF cycles and were chosen to meet the following criteria: (1) they were between 20 years old and 40 years old; (2) their IVF cycles had at least one embryo transfer; (3) they were in their first cycle; and (4) their IVF cycles used fresh nondonor eggs and embryos. All the women received ovarian stimulation using the gonadotropins available at the time of treatment. The stimulation method and dose were designed according to the age and ovarian function of each individual. The dosage of follicle-stimulating hormone was adjusted individually for seven days. Human chorionic gonadotropin (HCG, Profasi; Serono Laboratories, Randolph, MA, USA) (10,000 IU) was intramuscularly administered when three or more follicles were >16 mm at their largest diameters. Transvaginal follicular aspiration was performed 35–36 h later.

Follicular fluids were collected under the guidance of vaginal ultrasound, and then the follicles were rinsed with a fluid-flushing solution (flushing medium, MediCult, Denmark), and the follicular fluids of each patient were filtered and isolated. After follicle aspiration, granulosa cells were isolated from follicular fluids. The granulosa cells were washed and centrifuged at 250 g for 15 min at room temperature to remove blood cell contamination. The supernatant was removed, and the cell pellets were resuspended and gently placed on the top layer of Histopaque 1077 (Merck KGaA, Darmstadt, Germany) and centrifuged at 400 g for 30 min at room temperature. The fraction of the Histopaque 1077 interface was resuspended in 10 ml of phosphate-buffered saline (PBS) (Gibco, Thermo Fisher Scientific, Bleiswijk, NL) and centrifuged at 250 g for 15 min at room temperature. The cell pellets containing the granulosa cells were collected. The characterization of the granulosa and cumulus cells by flow cytometry enabled the blood cells in the follicular fluids to be easily removed, which was done with anti-human CD45 antibody-coated magnetic beads [52]. Moreover, we performed a positive selection marker of the FSH receptor, which is specifically expressed on the surface of cumulus cells, enabling their selection [53] and the distinguishing of granulosa and cumulus cells from blood cells.

### 4.2. Assessment of Hormones

During the IVF cycle, some monitoring was performed to ensure that the patient’s response to ovulation stimulation was in progress. The follicle sizes and numbers were measured by ultrasound. The patient’s blood was collected 10 min before the HCG injection to measure E2 levels, and 36 h after the HCG injection (before oocyte retrieval) to measure progesterone levels. The levels of E2 and P4 in the serum and follicular fluids were analyzed using radioimmunoassay (RIA) (Packard Cobra II Gamma Counter, Ramsey, MN, USA). 

### 4.3. Measurement of Mitochondrial Membrane Potential and Mitochondrial Mass

Granulosa cells were revealed by positive staining of the follicle-stimulating hormone receptor (FSHR). Granulosa cells were treated with a rabbit anti-FSHR primary antibody (cat# 3929, Sigma-Aldrich, Saint Louis, MO, USA) and then a goat anti-rabbit immunoglobulin G (IgG) secondary antibody (cat# ab6717, Abcam, Cambridge, UK). 

For the analysis of mitochondrial membrane potential, granulosa cells were concurrently treated with N, N, N’, N’-tetra-methylethylenediamine (TMRE) (cat# T669, Molecular probes, Life Technologies, Auckland, NZ) or MitoTracker Red FM (cat# M22425, Molecular probes) at 37 °C for 60 min, washed with PBS and centrifuged at 250 g for 15 min at room temperature to remove excess dye. For the analysis of mitochondrial mass, granulosa cells were stained with nonyl acridine orange (NAO) (cat# A1372 Molecular probes) at 37 °C for 30 min. Following staining and washing with PBS, the samples were centrifuged at 250 g for 15 min at room temperature to remove excess dye.

Flow cytometry analysis was performed using a FACSCalibur system (Becton, Dickinson and Company, BD Biosciences; USA) with the side-scattered light (SSC) detector voltage set at 415, the FL1 detector voltage set at 635, the FL2 detector voltage set at 614, and the FL3 detector voltage set at 150 voltage.

### 4.4. Assessment of Mitochondrial Morphology

The fluorescent staining was performed to assess the mitochondrial network by transfection with pLV-mitoDsRed (#44386, Addgene, Watertown, MA, USA) and to detect nuclei using 4’,6-diamidino-2-phenylindole (DAPI; Molecular Probes). Cells were fixed in 4% paraformaldehyde for 15 min and then incubated with DAPI for 3 min and mounted with Fluoromount (Sigma-Aldrich Corp.). Cells were mounted with Fluoromount-G™ (Thermo Fisher Scientific) and examined with a confocal fluorescence microscope (TCS SP5; Leica Microsystems CMS GmbH, Mannheim, Germany). Confocal fluorescent images were captured using the Leica SP5 confocal microscope fitted with an Apochromat 63 × 1.4 NA immersion objective with three lasers (argon, 488 nm; helium/neon, 543 nm; diode, 405 nm).

### 4.5. Protein Immunoblotting

Granulosa cells were lysed in 5 volumes of lysis buffer and then were centrifuged at 20,000 g for 30 min at 4 °C. The supernatant was collected. Protein levels were detected by Bio-Rad protein assay (cat# 500-0006; Bio-Rad Laboratories Inc, Hercules, CA). Protein samples from each infertile individual were subjected to 10% sodium dodecyl sulfate polyacrylamide gel electrophoresis (SDS-PAGE) and then were transferred to a polyvinylidene fluoride (PVDF) membrane (Hybond-P; GE Healthcare Bioscience, Fribourg, Switzerland). After the transfer was completed, the PVDF membrane was placed in a blocking buffer for 60 min. Then, the primary antibodies, rabbit anti-human StAR antibody (cat# ab3343, Abcam) and goat anti-human 3β-HSD antibody (cat# sc-30821, Santa Cruz), and then the secondary antibody anti-goat IgG-HRP (cat# sc-2020, Santa Cruz), were incubated with the membrane, which was followed by it being treated with enhanced chemiluminescence detection (ECL) using an ECL system (GE Healthcare Life Sciences, Chicago, IL, USA). Images were captured with ImageQuant LAS4000 (GE Healthcare Life Sciences). Densitometric measurements of immunoblotting bands were performed with the ImageJ program.

### 4.6. Analysis of Apoptosis

Apoptotic granulosa cells were identified by staining with Annexin V-fluorescein isothiocyanate (FITC) and Propidium iodide (PI) using a Vybrant^TM^ apoptosis assay kit (Molecular Probes, Invitrogen, Paisley, UK). Aliquots of 1 × 10^5^ cells were treated with 100 μl of binding buffer containing 1 μl of Annexin V-FITC and 10 μg/ml PI for 15 min, after which 400 μl of binding buffer was added. The flow cytometer was a FACSCalibur (Becton, Dickinson and Company, BD Biosciences; USA); its instrument parameters were set as follows—SSC detector: 415 voltage; FL1 detector: 635 voltage; FL2 detector: 614 voltage; and FL3 detector: 150 voltage. Cells that stained with neither PI nor Annexin V were considered viable cells. 

### 4.7. Statistical Analysis

Statistical analysis methods were used as follows. All experimental data are represented by the mean ± standard deviation (SD). Statistical analysis of the data was determined by unpaired T-tests or linear regression analysis. Linear regression analysis was used to determine the statistical significance of the relationship between estradiol levels and mitochondrial membrane potential, or between the level of estradiol and steroidogenic enzymes. A *p*-value of less than 0.05 was considered statistically significant.

## References

[1] Practice Committee of American Society for Reproductive Medicine (2013). Definitions of infertility and recurrent pregnancy loss: A committee opinion. Fertil. Steril..

[2] Practice Committee of the American Society for Reproductive Medicine (2015). Testing and interpreting measures of ovarian reserve: A committee opinion. Fertil. Steril..

[3] Committee on Gynecologic Practice (2015). Committee opinion no. 618: Ovarian reserve testing. Obstet. Gynecol..

[4] Smith S., Pfeifer S.M., Collins J.A. (2003). Diagnosis and management of female infertility. JAMA.

[5] Chattopadhyay A.B., Rath S.K. (1999). Understanding male factor in infertility. Med. J. Armed Forces India.

[6] Niederberger C. (2001). WHO manual for the standardized investigation, diagnosis and management of the infertile male. Urology.

[7] Farquhar C. (2007). Endometriosis. BMJ.

[8] Brosens I., Benagiano G. (2011). Endometriosis, a modern syndrome. Indian J. Med. Res..

[9] Lagana A.S., Garzon S., Gotte M., Vigano P., Franchi M., Ghezzi F., Martin D.C. (2019). The pathogenesis of endometriosis: Molecular and cell biology insights. Int. J. Mol. Sci.

[10] Vercellini P., Vigano P., Somigliana E., Fedele L. (2014). Endometriosis: Pathogenesis and treatment. Nat. Rev. Endocrinol..

[11] Brosens I.A., Puttemans P.J., Deprest J. (1994). The endoscopic localization of endometrial implants in the ovarian chocolate cyst. Fertil. Steril..

[12] Zhang J., Bao Y., Zhou X., Zheng L. (2019). Polycystic ovary syndrome and mitochondrial dysfunction. Reprod. Biol. Endocrinol..

[13] Teede H.J., Misso M.L., Costello M.F., Dokras A., Laven J., Moran L., Piltonen T., Norman R.J., International P.N. (2018). Recommendations from the international evidence-based guideline for the assessment and management of polycystic ovary syndrome. Fertil. Steril..

[14] Uyar A., Torrealday S., Seli E. (2013). Cumulus and granulosa cell markers of oocyte and embryo quality. Fertil. Steril..

[15] Wyndham N., Marin Figueira P.G., Patrizio P. (2012). A persistent misperception: Assisted reproductive technology can reverse the aged biological clock. Fertil. Steril..

[16] Cakmak H., Franciosi F., Zamah A.M., Cedars M.I., Conti M. (2016). Dynamic secretion during meiotic reentry integrates the function of the oocyte and cumulus cells. Proc. Natl. Acad. Sci. USA.

[17] Hillier S.G., Whitelaw P.F., Smyth C.D. (1994). Follicular oestrogen synthesis: The two-cell, two-gonadotrophin model revisited. Mol. Cell. Endocrinol..

[18] Ting A.Y., Xu J., Stouffer R.L. (2015). Differential effects of estrogen and progesterone on development of primate secondary follicles in a steroid-depleted milieu in vitro. Hum. Reprod..

[19] Hamel M., Dufort I., Robert C., Gravel C., Leveille M.C., Leader A., Sirard M.A. (2008). Identification of differentially expressed markers in human follicular cells associated with competent oocytes. Hum. Reprod..

[20] Wathlet S., Adriaenssens T., Segers I., Verheyen G., Janssens R., Coucke W., Devroey P., Smitz J. (2012). New candidate genes to predict pregnancy outcome in single embryo transfer cycles when using cumulus cell gene expression. Fertil. Steril..

[21] Feuerstein P., Cadoret V., Dalbies-Tran R., Guerif F., Bidault R., Royere D. (2007). Gene expression in human cumulus cells: One approach to oocyte competence. Hum. Reprod..

[22] Miller W.L. (2017). Disorders in the initial steps of steroid hormone synthesis. J. Steroid. Biochem. Mol. Biol..

[23] Miller W.L. (2013). Steroid hormone synthesis in mitochondria. Mol. Cell. Endocrinol..

[24] Allen J.A., Shankara T., Janus P., Buck S., Diemer T., Hales K.H., Hales D.B. (2006). Energized, polarized, and actively respiring mitochondria are required for acute Leydig cell steroidogenesis. Endocrinology.

[25] Artemenko I.P., Zhao D., Hales D.B., Hales K.H., Jefcoate C.R. (2001). Mitochondrial processing of newly synthesized steroidogenic acute regulatory protein (StAR), but not total StAR, mediates cholesterol transfer to cytochrome P450 side chain cleavage enzyme in adrenal cells. J. Biol. Chem..

[26] Au H.K., Lin S.H., Huang S.Y., Yeh T.S., Tzeng C.R., Hsieh R.H. (2005). Deleted mitochondrial DNA in human luteinized granulosa cells. Ann. N. Y. Acad. Sci..

[27] Von Mengden L., Klamt F., Smitz J. (2020). Redox biology of human cumulus cells: Basic concepts, impact on oocyte quality, and potential clinical use. Antioxid. Redox Signal..

[28] Karuputhula N.B., Chattopadhyay R., Chakravarty B., Chaudhury K. (2013). Oxidative status in granulosa cells of infertile women undergoing IVF. Syst. Biol. Reprod. Med..

[29] Hsu A.L., Townsend P.M., Oehninger S., Castora F.J. (2015). Endometriosis may be associated with mitochondrial dysfunction in cumulus cells from subjects undergoing in vitro fertilization-intracytoplasmic sperm injection, as reflected by decreased adenosine triphosphate production. Fertil. Steril..

[30] Hoshino Y. (2018). Updating the markers for oocyte quality evaluation: Intracellular temperature as a new index. Reprod. Med. Biol..

[31] Tanghe S., Van Soom A., Nauwynck H., Coryn M., de Kruif A. (2002). Minireview: Functions of the cumulus oophorus during oocyte maturation, ovulation, and fertilization. Mol. Reprod. Dev..

[32] Huang Z., Wells D. (2010). The human oocyte and cumulus cells relationship: New insights from the cumulus cell transcriptome. Mol. Hum. Reprod..

[33] Boucret L., Chao de la Barca J.M., Moriniere C., Desquiret V., Ferre-L’Hotellier V., Descamps P., Marcaillou C., Reynier P., Procaccio V., May-Panloup P. (2015). Relationship between diminished ovarian reserve and mitochondrial biogenesis in cumulus cells. Hum. Reprod..

[34] Senapati S., Sammel M.D., Morse C., Barnhart K.T. (2016). Impact of endometriosis on in vitro fertilization outcomes: An evaluation of the Society for Assisted Reproductive Technologies Database. Fertil. Steril..

[35] Harb H.M., Gallos I.D., Chu J., Harb M., Coomarasamy A. (2013). The effect of endometriosis on in vitro fertilization outcome: A systematic review and meta-analysis. BJOG.

[36] Coccia M.E., Rizzello F., Mariani G., Bulletti C., Palagiano A., Scarselli G. (2011). Impact of endometriosis on in vitro fertilization and embryo transfer cycles in young women: A stage-dependent interference. Acta Obstet. Gynecol. Scand..

[37] Suzuki T., Izumi S., Matsubayashi H., Awaji H., Yoshikata K., Makino T. (2005). Impact of ovarian endometrioma on oocytes and pregnancy outcome in in vitro fertilization. Fertil. Steril..

[38] Opoien H.K., Fedorcsak P., Omland A.K., Abyholm T., Bjercke S., Ertzeid G., Oldereid N., Mellembakken J.R., Tanbo T. (2012). In vitro fertilization is a successful treatment in endometriosis-associated infertility. Fertil. Steril..

[39] Zamah A.M., Hassis M.E., Albertolle M.E., Williams K.E. (2015). Proteomic analysis of human follicular fluid from fertile women. Clin. Proteomics.

[40] Rodgers R.J., Irving-Rodgers H.F. (2010). Formation of the ovarian follicular antrum and follicular fluid. Biol. Reprod..

[41] Sanchez A.M., Vanni V.S., Bartiromo L., Papaleo E., Zilberberg E., Candiani M., Orvieto R., Vigano P. (2017). Is the oocyte quality affected by endometriosis? A review of the literature. J. Ovarian Res..

[42] Ishihara Y., Takemoto T., Ishida A., Yamazaki T. (2015). Protective actions of 17beta-estradiol and progesterone on oxidative neuronal injury induced by organometallic compounds. Oxid. Med. Cell. Longev..

[43] Lamb J.D., Zamah A.M., Shen S., McCulloch C., Cedars M.I., Rosen M.P. (2010). Follicular fluid steroid hormone levels are associated with fertilization outcome after intracytoplasmic sperm injection. Fertil. Steril..

[44] Carpintero N.L., Suarez O.A., Mangas C.C., Varea C.G., Rioja R.G. (2014). Follicular steroid hormones as markers of oocyte quality and oocyte development potential. J. Hum. Reprod. Sci..

[45] Jones H.W., Acosta A., Andrews M.C., Garcia J.E., Jones G.S., Mantzavinos T., McDowell J., Sandow B., Veeck L., Whibley T. (1983). The importance of the follicular phase to success and failure in in vitro fertilization. Fertil. Steril..

[46] Fisher S., Grin A., Paltoo A., Shapiro H.M. (2005). Falling estradiol levels as a result of intentional reduction in gonadotrophin dose are not associated with poor IVF outcomes, whereas spontaneously falling estradiol levels result in low clinical pregnancy rates. Hum. Reprod..

[47] Segawa T., Teramoto S., Omi K., Miyauchi O., Watanabe Y., Osada H. (2015). Changes in estrone and estradiol levels during follicle development: A retrospective large-scale study. Reprod. Biol. Endocrinol..

[48] Nebert D.W., Wikvall K., Miller W.L. (2013). Human cytochromes P450 in health and disease. Philos. Trans. R. Soc. Lond. B Biol. Sci..

[49] Prasad M., Thomas J.L., Whittal R.M., Bose H.S. (2012). Mitochondrial 3beta-hydroxysteroid dehydrogenase enzyme activity requires reversible pH-dependent conformational change at the intermembrane space. J. Biol. Chem..

[50] Castillo A.F., Orlando U., Helfenberger K.E., Poderoso C., Podesta E.J. (2015). The role of mitochondrial fusion and StAR phosphorylation in the regulation of StAR activity and steroidogenesis. Mol. Cell. Endocrinol..

[51] Regan S.L.P., Knight P.G., Yovich J.L., Leung Y., Arfuso F., Dharmarajan A. (2018). Granulosa cell apoptosis in the ovarian follicle-a changing view. Front. Endocrinol..

[52] Schreier S., Sawaisorn P., Udomsangpetch R., Triampo W. (2017). Advances in rare cell isolation: An optimization and evaluation study. J. Transl. Med..

[53] Vigone G., Merico V., Redi C.A., Mazzini G., Garagna S., Zuccotti M. (2015). FSH and LH receptors are differentially expressed in cumulus cells surrounding developmentally competent and incompetent mouse fully grown antral oocytes. Reprod. Fertil. Dev..

